# Snake Venom: A Promising Source of Neurotoxins Targeting Voltage-Gated Potassium Channels

**DOI:** 10.3390/toxins16010012

**Published:** 2023-12-25

**Authors:** Altaf K. AlShammari, Tarek Mohamed Abd El-Aziz, Ahmed Al-Sabi

**Affiliations:** 1 College of Engineering and Technology, American University of the Middle East, Egaila 54200, Kuwait; 39707@aum.edu.kw; 2 Zoology Department, Faculty of Science, Minia University, El-Minia 61519, Egypt; 3 Department of Cellular and Integrative Physiology, University of Texas Health Science Center at San Antonio, San Antonio, TX 78229, USA

**Keywords:** BPTI-Kunitz polypeptides, CRISPs, dendrotoxins, Kv channels blockers, PLA_2_ neurotoxins, presynaptic neurotoxins, SVSPs, snake venom

## Abstract

The venom derived from various sources of snakes represents a vast collection of predominantly protein-based toxins that exhibit a wide range of biological actions, including but not limited to inflammation, pain, cytotoxicity, cardiotoxicity, and neurotoxicity. The venom of a particular snake species is composed of several toxins, while the venoms of around 600 venomous snake species collectively encompass a substantial reservoir of pharmacologically intriguing compounds. Despite extensive research efforts, a significant portion of snake venoms remains uncharacterized. Recent findings have demonstrated the potential application of neurotoxins derived from snake venom in selectively targeting voltage-gated potassium channels (Kv). These neurotoxins include BPTI-Kunitz polypeptides, PLA_2_ neurotoxins, CRISPs, SVSPs, and various others. This study provides a comprehensive analysis of the existing literature on the significance of Kv channels in various tissues, highlighting their crucial role as proteins susceptible to modulation by diverse snake venoms. These toxins have demonstrated potential as valuable pharmacological resources and research tools for investigating the structural and functional characteristics of Kv channels.

## 1. Introduction

Throughout a span of over 120 million years, poisonous snakes belonging to the Order Squamata; namely, the suborder Serpentes, have developed a diverse array of venoms containing bioactive molecules. These snakes, which encompass approximately 600 species and are classified under the Elapidae, Viperidae, and Colubridae families, have undergone significant evolutionary adaptations in their venom composition [1]. These compounds exhibit a range of actions that target significant physiological pathways and organs, encompassing cytotoxic, neurotoxic, cardiotoxic, myotoxic, and other enzymatic activities [2,3]. Hence, snake envenomation poses a considerable health risk in numerous regions throughout. According to estimates, a total of 7400 individuals experience snake bites on a daily basis, resulting in an annual fatality count ranging from 81,000 to 138,000 [4,5]. Moreover, this unfortunate outcome leaves over 400,000 individuals with enduring bodily or psychological impairments, such as blindness, amputation, and post-traumatic stress disorder [4]. Snake venoms comprise intricate combinations of diverse chemicals, encompassing peptides and proteins, which serve as protective mechanisms, aid in prey acquisition, and/or discourage competition [2]. Nevertheless, several toxins derived from snake venom have demonstrated promising applications as diagnostic tools, therapeutic agents, or pharmacological candidates [3]. Through recent advanced in research and drug discovery, snake venom components gain in-depth attention as invaluable sources of therapeutics in many medical fields from antimicrobial to anti-cancer research, to name a few [6,7,8,9,10,11,12,13].

Potassium (Kv) channels are found in the membranes of numerous cell types due to their diverse physiological significance [14,15]. These potassium channels are tetrameric glycoproteins which have the ability to form pores through the membranes that selectively allow the passage of potassium ions in response to alterations in the voltage of the membrane. Moreover, they exhibit rapid activation and inactivation of potassium currents when the cell membrane is depolarized [16,17]. The study of animal peptide toxins, such as those found in snake venom, and their interaction with Kv channels has yielded significant insights into the physiological properties of potassium (K^+^) channels. These compounds are predominantly found in the venoms of several species, including sea anemones, spiders, scorpions, honeybees, and snails belonging to the *Conus* genus [18,19]. These substances have well-established modes of action [20]. Extensive examination of the structures and functionalities of many animal toxins has yielded numerous advantages, notably the development of medicines derived from snake toxins [18]. The modification of their chemical groups can lead to the acquisition of therapeutic properties through the manipulation of pharmacological selectivity, specificity, and potency [21,22]. Moreover, these invaluable polypeptides can serve as experimental resources for investigating biological phenomena. This review aims to consolidate the existing research on the structural and functional characteristics of peptide neurotoxins derived from snake venom, specifically focusing on their role as modulators for K^+^ channels. The purpose of this study is to uncover the pharmacological characteristics and address the lack of understanding regarding the significance of these peptides in fundamental biomedical research and the development of new drugs. Initially, an overview was provided on the structure and medicinal significance of their targets, the Kv channels. Subsequently, the investigation focuses on the protein categories of neurotoxins present in snake venoms; namely, BPTI-Kunitz type, PLA_2_, CRISPs, SVSPs, and the three-finger toxins. Subsequently, the scientific inquiries for each toxin were examined by considering their protein family structures and pharmacological discoveries as outlined in thorough and up-to-date literature. Ultimately, we presented the efforts of scientists in using neurotoxins generated from snake venom as potential therapeutics attributes for chronic Kv channelopathies.

## *2.* Voltage-Gated Potassium (Kv) Channels

Potassium channels are the most extensive and heterogeneous group of ion channels. The voltage-gated potassium channels (Kv) are the most significant subfamily among K^+^ channels. Potassium ions play a crucial role in various physiological processes, as they facilitate the controlled movement of ions along the electrochemical gradient [23]. These processes include the regulation of excitability and the modulation of neuronal action potentials [24,25], as well as the facilitation of muscular contraction [26,27,28] and the regulation of calcium signaling pathways, among others [29]. These functions have been extensively reviewed by previous studies [14,15]. The genes responsible for encoding Kv channel α-subunits have been identified and can be categorized into twelve distinct subfamilies [15]. These subfamilies include Kv1 (Shaker) with eight members, Kv2 (Shab) with two members, Kv3 (Shaw) with four members, Kv4 (Shal) having five members, Kv7 (KvLQT) with five members, Kv10 (HERG) with two members, Kv11 (also known as EAG) with three members, and Kv12 (ELK) with three members, as well as the modulatory subfamilies Kv5 (consisting of one member), Kv6 (consisting of four members), Kv8 (consisting of two members), and Kv9 (consisting of three members). This classification system has been established based on the identified genes for Kv channel α-subunits. These channels have been found to be involved in a wide range of neurological, cardiac, and immunological illnesses, making them significant targets for therapeutic interventions [30].

**Figure 1:**
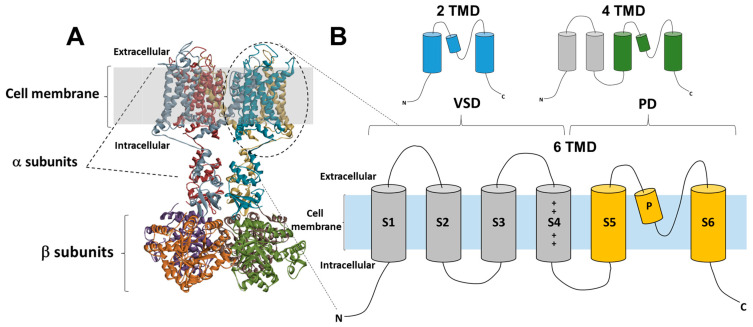
The structure of Kv channels. (**A**) Schematic side-view presentation of a Kv1.2-2.1 chimeric channel obtained from a single-particle cryo-EM structure (PDB: 6EBK, [38]) showing the four α and four β auxiliary subunits. Shaded areas are the cell membrane. The image was generated using BIOVIA Discovery Studio Visualizer software (v21.1.0). (**B**) Schematic illustration of Kv channel membrane topology (two, four, or six transmembrane domains; TMDs). The majority of Kv channels have six TM domains. Here, the side-view depicts one of the four transmembrane α subunits: each subunit includes the voltage-sensing domain (in gray, S1–S4: VSD) and the pore domain between S5 and S6 segments (the loop between orange parts: PD).

The number of transmembrane domains (TMD) observed in K^+^ channels has been determined through genetic and structural investigation, revealing the presence of two, four, or six TMD. The majority of Kv channels exhibit a six-TMD arrangement in their functional assembly (see Figure 1A). The regulation of the pore opening is governed by the voltage-sensing domain, which is composed of transmembrane segments S1–S4. This domain, also known as the voltage-sensor domain (VSD), is coupled to the pore domain (PD) through the intracellular loop between segments S4 and S5 [31] (Figure 1B). The latter domain consists of transmembrane proteins S5 and S6, which feature a re-entrant pore loop containing the K^+^ selectivity motif TVGYG [32]. Following the repolarization phase, the voltage-sensitive domain (VSD) undergoes deactivation, wherein the channel gate is closed, thereby impeding the passage of ions and restoring the VSD to its original resting state. Channels have the ability to be revived, however, if the stimulation caused by depolarization lasts longer than a few milliseconds, inactivation occurs and stops the permeability of potassium ions. Kv channels undergo recovery from the inactivation state exclusively following a short duration at a hyperpolarized potential [33]. C-type inactivation, also known as slow inactivation, is occurring after tens or hundreds of milliseconds from channel activation and is observed in the majority of Kv rectifying channels [34]. Recent research findings provide evidence for a mechanism in which the reorganization of amino acids within the inner cavity and outer vestibule of a channel is accompanied by the redistribution of structural water molecules. This process ultimately results in the collapse of the permeation pathway in C-type inactivation. This mechanism has been extensively discussed and reviewed in Ref. [35]. Several types of Kv channels exhibit rapid inactivation or N-type inactivation, which happens shortly after channel activation. This inactivation is primarily caused by an intracellular blockage by the channel’s intracellular N-terminus, also known as the inactivation particle [36,37]. 

Functional Kv1 channels consist of four α subunits and four cytoplasmic auxiliary Kvβ subunits [39,40] (Figure 1B) once they are expressed as a cell membrane molecule. Previous research has demonstrated that in heterologous expression systems, Kv1 α and Kvβ subunits have the ability to form both homo- and heteromeric complexes in a promiscuous manner [39,41]. The generation of functional Kv1 channels is attributed to the diverse combinations of eight distinct subtypes of Kv1 α subunits and three subtypes of Kvβ subunits expressed in the mammalian brain. These subunits exhibit unique biophysical and pharmacological features, as discussed in a comprehensive study [15]. Nevertheless, Kv1 channels derived from the mammalian brain display a restricted range of subunit composition [42,43].

## 3. Snake Venom

The composition of snake venoms is characterized by a complex mixture of peptides, proteins, and non-protein components [2,44]. Despite the variations seen among different snake species and their geographical distribution, it is noteworthy that a significant proportion of the protein component in snakes exhibits enzymatic activity. The pro-inflammatory effects of the venom are commonly ascribed mostly to metalloproteases (SVMPs) and phospholipases A_2_ (PLA_2_s) [2,3]. Conversely, non-enzymatic proteins exacerbate the intensity of the envenomation. The venoms produced by snakes have the ability to selectively target particular receptors, ion channels, or plasma proteins, or extracellular components, thereby functioning as agonists, antagonists, or modulators. The pharmaceutical effects described would disrupt the individual’s physiological processes, leading to a variety of hazardous outcomes [45]. Included in this group of proteins, besides SVMPs and PLA_2_s, are disintegrins, C-type lectins, three-finger toxins, bradykinin-potentiating peptides (BPPs), Kunitz-type polypeptide, cysteine-rich secretory proteins (CRISPs), serpins, ICK peptides, and serine proteases (SVSPs) [9]. The components that specifically target Kv channels aim to disrupt the normal functioning of these channels, resulting in a decrease in the excitability of the affected tissues and ultimately leading to paralysis in the individual. In this discourse, we shall incorporate the most recent Kv blockers and modulators inside their respective group classifications.

### 3.1. Polypeptides of the BPTI-Kunitz Type

#### 3.1.1. Dendrotoxins 

During the 1980s, a collection of seven peptide-based inhibitors targeting Kv channels was obtained from the venoms of specific snake species. These species include the green mamba (*Dendroaspis angusticeps*), which yielded αDTX, βDTX, γDTX, and δDTX; the black mamba (*D. polylepis*), which provided DTX-I and DTX-K; and *D. viridis* snakes, which contributed DV14 [46,47,48,49,50], along with DaE from *D. angusticeps* [51]. The facilitation of neurotransmission release at neuronal synapses in the peripheral and central nervous systems has been demonstrated through the inhibition of neuronal Kv channels’ K^+^ currents by dendrotoxins (DTXs) [52,53]. The existing sequence data can be categorized into two distinct subfamilies, as indicated in Table 1, exhibiting an approximate amino acid similarity of 60%. The partial sequencing of βDTX and γDTX, both belonging to the DTX family, has been conducted. However, the subfamily classification of these sequences remains uncertain [50,54]. As evidenced by multiple scholarly works (refer to Table 1), the predominant neuronal Kv1.1 and 1.2 channels are selectively suppressed by dendrotoxins. It is worth mentioning that the DTXs constitute only a portion of the protein constituents found in mambas. These constituents include Calcicudines and Calciseptines, which function as blockers of Cav channels, as well as protease inhibitors, muscarinic toxins, α-neurotoxins, and anticholinesterase, among others [55]. The combined impact of all venom components renders these naturally occurring substances a rich resource for pharmacological investigations.

#### The Molecular Structures of Dendrotoxins 

**Figure 2:**
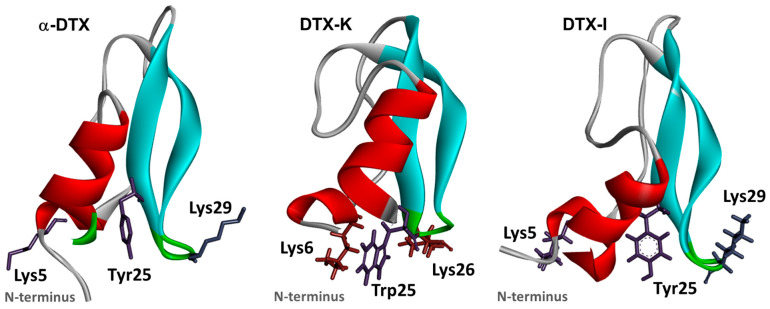
Dendrotoxins. Surface model representatives of αDTX from *D. angusticeps* (PDB: 1DTX; [61]), DTX-I from *D. polylepis* (PBD: 1DEM; [62]), and DTX-K from *D. polylepis* (PBD: 1DTK; [63]). The colored parts reflect the secondary constrained structures of the peptides (red for the α-helices, cyan for β-pleated sheets, and green for the loops). Some of the representative equivalent key residues of these toxins are shown at the N-termini and the β-hairpin regions (the cationic domains), for comparison. The C-termini are located behind the structures. The molecular structures were visualized using Biovia Discovery Studio Visualizer (v21.1.0).

DTXs are characterized by their structural composition, which consists of single-chain polypeptides with a relatively low molecular mass of ~7 kDa. These polypeptides are typically 57 to 60 amino acid residues long and include three conserved disulfide bridges, as depicted in Figure 2. DTX-K comprises three structural elements: a 3_10_-helix spanning from the 3rd to the 7th amino acid residues, a β-hairpin composed of the region between the 18th to the 35th, and an α-helix between the 47th to the 56th residue [60]. The DTXs exhibit sequence homology with Kunitz serine protease inhibitors, such as the bovine pancreatic trypsin inhibitor (BPTI) (Table 1). However, the toxins with the highest potency have minimal activity as protease inhibitors, and these inhibitors do not effectively inhibit K^+^ channels [52,54].

#### The Pharmacology of Dendrotoxins 

When administered via the intra-cerebroventricular route, DTXs have strong convulsant properties by crossing the blood–brain barrier (BBB) and inducing an elevation in activity at monoaminergic terminals, even at very low concentrations measured in nanograms per gram of body weight [64]. The administration of 35 pmol of dendrotoxin K (DTX-K) and α-dendrotoxin (αDTX) directly into the hippocampus resulted in an increase in extracellular levels of aspartate and glutamate [65]. At the neuromuscular junctions of mice and frogs, these toxins enhance the quantal content and elicit single nerve action potentials, resulting in brief episodes of repeated activity. The observed temporary functional shift was in line with an effect on presynaptic Kv channels [65]. 

Different potencies of DTXs have been observed to block both slowly inactivating and non-inactivating Kv currents in peripheral sensory neurons of rats. The δ-dendrotoxin has a relatively high degree of selectivity towards non-inactivating Kv currents, with an IC_50_ value of 0.24 nM. On the other hand, the α-dendrotoxin demonstrates more potency in inhibiting slowly inactivating Kv currents, with an IC_50_ value of 1 nM [66,67]. In a sequence of experiments including toxin binding and affinity chromatography, the purification of a specific group of Kv channels derived from the mammalian brain was achieved. Through cloning, it was determined that these channels belong to the Kv1 family. These findings underscore the significance of DTXs as blockers and valuable instruments for investigating these particular targets [52]. Subsequent investigations have provided more evidence indicating that DTXs exhibit exceptional potency, specificity, and extensive research attention as polypeptides derived from snake venom. Their activity is limited to a small number of Kv1 α subunits; namely, Kv1.1, 1.2, and 1.6 (as presented in Table 1). 

The residues that play a crucial role in determining the potency of DTXs against Kv1 channels have been thoroughly characterized. The channel blocking of αDTX was found to be dependent on specific amino acid residues at the N-terminus region, including Arg1, Arg2, Lys3, Leu4, Lys5, Ile6, and Leu7, as determined through a mutational analysis [68]. In a subsequent investigation, the functional regions of αDTX that play a crucial role in its interaction with Kv1 channels on synaptosomal membranes in the rat brain have been identified. These regions encompass six specific binding residues located in the N-terminal region; namely, Arg3, Arg4, Lys5, Leu6, Ile8, and Leu9. Notably, among these residues, Lys5 and Leu9 exhibit the highest significance in this binding process [69]. The interaction between DTX-K and Kv1.1 channels has been investigated using site-directed mutagenesis studies, revealing that Lys3, Tyr4, Lys6, Pro8, Arg10, Trp25, Lys26, and Lys28 are the primary residues implicated [60,70] (Figure 2). The findings of these investigations indicate that DTXs primarily utilize N-terminal residues for the recognition of Kv1 potassium channels [71].

**Figure 3:**
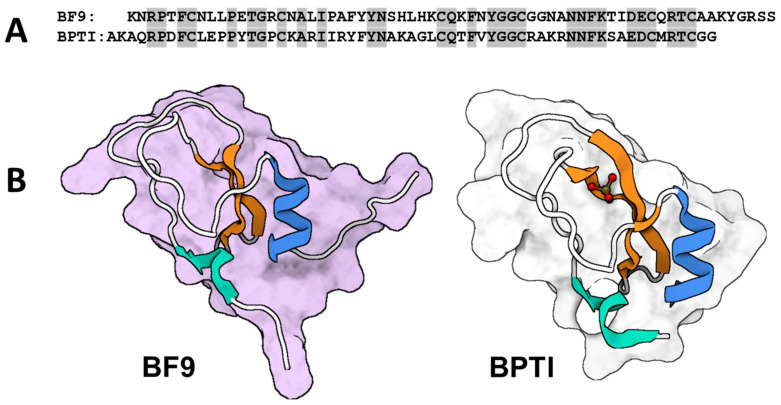
The sequence and the 3D molecular structure of BF9 compared with those of the BPTI (**A**) Amino acid sequences of BF9 and homologs alignment of BPTI. Shaded areas indicate identical sequence alignments. (**B**) Surface model representatives of BF9 from *Bungarus fasciatus* snake (PDB: 1JC6; [82]) and BPTI (PDB: 6PTI; [80]). The colored parts reflect the secondary constrained structures of the peptides (cyan and blue for α-helices and orange for β-pleated sheets). The molecular structure was visualized by Biorender.com, accessed on 9 November 2023.

The presence of conserved patterns of disulfide bridging [60] may account for the observed similarity in the backbones of the DTXs and serine protease inhibitors, despite their distinct structural variations (as depicted in Figure 2 and Figure 3). Both groups of homologs had a cationic domain containing positively charged residues situated in the lower region of their respective structures. The formation of this domain is facilitated by the inclusion of both the N- and C-termini, as well as residues 27–30 of the β-turn [56]. The results of these experiments indicate that the cationic domain of DTX-K exhibits an interaction with a region of negative charge located on the channel; namely, at the extracellular turret region of the PD. An investigation was conducted on the impact of alanine-substitution mutagenesis on DTX-K, specifically targeting the positively charged lysine residues (Lys24, 26, and 28) located inside the hairpin structure. The results revealed a reduction in binding affinity, with the Lys26 mutant exhibiting the most significant decrease. In the aforementioned study, it was shown that the introduction of a mutation (Trp25Ala) resulted in a decrease in binding affinity. This finding indicates that both positively charged and hydrophobic residues present in the β-hairpin play a crucial role in the interaction between the toxin and the channel. The combined findings of these investigations indicate that the 3_10_-helix and β-hairpin domains play a significant role in the interaction of the channel, as modifications to certain residues within these structural features result in a decrease in binding affinity [45,55]. Subsequent research on DTX-I substantiated that the binding of the toxin is attributed to residues located in both the N-terminus and certain residues towards the C-terminus (see Figure 2; Ref. [56]). 

The presence of a functional dyad or triad of residues in a particular toxin is of utmost importance for its ability to bind to channels, as it has been observed in toxins derived from various sources [20]. Two potential triads were found in DTXs; namely, Lysine 19, Tyr17, and Trp37, as well as Lys at positions 5, 28, and 29 (refer to Figure 2). In contrast, the binding of αDTX, which is the homolog of DTX-I from *D. angusticeps* as indicated in Table 1, is solely reliant on residues located at the N-terminus for its binding capability. Conversely, the residues found in the β-hairpin region, specifically the DTX-I Lys19/Tyr17/Trp37 triad, do not play a significant role in the binding process. Indeed, the presence of three distinct amino acid residues at positions 18, 34, and 36 in αDTX and DTX-I leads to significant alterations in the electrostatic potential of the domain. Therefore, it is hypothesized that the presence of acidic residues in the vicinity of Lys19 in αDTX may disrupt the interactions between Lys19 and negatively charged residues within the pore of the Kv1 channel [56]. It has been proposed that the differential selectivity of DTX-K, which exclusively blocks Kv1.1 channels, compared to αDTX, which inhibits Kv1.1, 1.2, and 1.6 channels, may be attributed to the presence of two critical residue regions in DTX-K (located in the β-hairpin and the 3_10_-helix), as opposed to only one region in αDTX (located at the N-terminus and not the β-hairpin). This suggests that a larger portion of the DTX-K toxin molecule interacts with the channel pore, necessitating a higher level of specificity in the pore sequence. This information is summarized in Table 1 (Refs. [69,70]). 

The findings of a research investigation examining the interactions between δDTX and the Shaker K^+^ channel, whereby the pore area was genetically altered to mimic that of Kv1.1 [72], revealed that a specific triangle cluster consisting of seven amino acids constituted the binding interface for the toxin. The proposed hypothesis put forth by the authors suggests that the asymmetric ligand exhibits off-center binding within the pore. This binding primarily involves interaction with the turret region of one α subunit, while also contacting the two adjacent subunits. Consequently, the ligand does not engage in equal interaction with all four subunits. Based on the assumption of a one-to-one interaction between a toxin and a channel, the authors propose two potential explanations for the inhibitory mechanism of the channels. Firstly, they suggest that the positively charged toxin may function as a repellant for K^+^ ions. Alternatively, they propose that the attachment of a DTX molecule to a turret could lead to a structurally more rigid pore, thereby exerting a negative influence on ion flow. This hypothesis is considered to be more plausible [57]. 

#### The Importance of Dendrotoxins in Targeting Neuronal Kv1 Channels

The evaluation of the responsiveness of homomeric and heteromeric Kv1 channels to selective DTX substantiates the specificity of these toxins towards various combinations of these endogenous channels in both normal physiological conditions and pathological disorders. DTX-K demonstrated high potency and selectivity in blocking exclusively the Kv1.1 homo-tetrameric channels, as evidenced by its sub-nanomolar IC_50_ [66,73]. In these studies, the incorporation of a solitary Kv1.2 subunit into the tetrameric structure resulted in a reduction in the vulnerability of the heteromeric channel to blocking by DTX-K. However, the insensitivity of the concatemers of channels to DTX-K was seen when two or four copies of Kv1.2 subunits were present. The findings presented in this study provide evidence in favor of the previously suggested hypothesis that the asymmetric ligand binds in an off-center manner to the pore, resulting in minimal interaction with two neighboring subunits.

The strong binding affinity of DTXs to presynaptic Kv1 channels demonstrates significant promise for a wide range of applications. One potential candidate for cancer therapy is DTX-K, which shows promise as a toxin. According to a study, it was revealed that there was a significant upregulation of the Kv1.1 channel in individuals diagnosed with cervical cancer. This upregulation was found to be associated with a negative prognosis. Furthermore, in laboratory experiments conducted on HeLa cells [74], the inhibition of this channel resulted in a decrease in cell proliferation. In a separate investigation, it was discovered that DTX-K had the ability to inhibit the growth of lung adenocarcinoma in vivo [75], as well as decrease the proliferation of non-small cell lung cancer cells that were resistant to chemotherapy, both in vitro and in vivo [76]. These findings, along with anticipated future discoveries, suggest a promising potential for Kv1 channels in the context of cancer and the use of DTX-K to investigate this disease and explore potential therapeutic interventions. Furthermore, it is worth noting that DTXs have the capability to accurately measure the synaptic density within the central nervous system (CNS). The application of this prospective utilization could be extended to the diagnosis of neurodegenerative disorders, such as the identification of synaptic loss in hippocampal tissue using α-DTX [77].

The Kv1.1 null mutants demonstrate increased excitability and enhanced axonal conductivity [78,79], indicating that Kv1.1-containing channels have a significant inhibitory effect on demyelinated neurons. The study conducted by [73] revealed the occurrence of over-expression of Kv1.1 subunits and the subsequent development of aberrant homomeric Kv1.1 channels in the demyelinated optic nerve (ON) axons of mice that were administered cuprizone. Given the observed elevation and aberrant manifestation of the Kv1.1 subunit in demyelinated axons of the ON, as well as the subsequent restoration of neuronal conduction to near-normal levels with the administration of DTX-K, it is plausible to consider the Kv1.1 channel as a promising target for interventions aimed at ameliorating the condition. The interference of 4-AP, which is considered one of the most promising possibilities, with the process of remyelination and regeneration of damaged oligodendrocytes [73] presents challenges in its clinical application for the purpose of restoring axonal conductivity. This statement underscores the imperative nature of promptly identifying and assessing new potential pharmaceutical agents.

#### 3.1.2. BF9

Bioinformatics and biological assays were used to characterize a novel polypeptide from the Kunitz-type superfamily [80,81]. The peptide toxin denoted as BF9 (Figure 3), was extracted from the *Bungarus fasciatus* snake and is composed of 65 amino acid residues, which are connected by three disulfide bridges [82]. Upon comparison, it is evident that the three-dimensional conformation of BF9 resembles the bovine pancreatic trypsin inhibitor (BPTI) proteins in terms of secondary structural elements. However, notable distinctions are observed in the loop regions and beta-turn between the two. According to Chen et al.’s findings, the biological functions of proteins are associated with the flexibility, rigidity, and variability of amino acid residues in both the loop and beta-turn regions [81]. It is noteworthy that BF9 has been found to exhibit bifunctional properties, acting as an inhibitor for both α-chymotrypsin and the Kv1.3 channel. This was demonstrated by Chen et al. [82] and further supported by Yang et al. [81], who reported an IC_50_ value of 120 nM for the inhibition of the Kv1.3 channel by BF9. In their comprehensive study, Yang and colleagues conducted a thorough investigation which revealed that specific residues, namely, Lys1, Arg55, Lys60, and Lys63, within the BF9 protein may employ an abundance of basic residues positioned at the N- and C-terminal regions. This molecular mechanism enables BF9 to effectively interact with Kv1.3 channels. The postulated mechanism of action for the “Basic Lid” is similarly observed in other potassium channel blockers [20]. The distinctive merging of Kv channel and serine protease inhibitory characteristics offers a fresh perspective on snake Kunitz-type peptides’ divergent evolution and functional implications.

### 3.2. Phospholipase A_2_ Neurotoxins

Secreted phospholipases A_2_ (sPLA_2_) represent a class of enzymes that exhibit structural similarities and are responsible for catalyzing the hydrolysis of glycerophospholipids derived from cell membranes at the sn-2 position. This enzymatic process results in the liberation of free fatty acids and lysophospholipids [83]. The majority of sPLA_2_s have comparable structural characteristics, such as a relatively small molecular mass ranging from 13 to 15 kDa, a compact configuration featuring typically seven (occasionally six or eight) disulfide bonds in the single-chain polypeptides, and a conserved catalytic activity that is dependent on the presence of calcium ions [84,85]. Various organisms, including insects, snake venoms, mammalian tissues, plants, bacteria, fungi, and viruses, have been demonstrated to possess these substances [83]. Snake venom PLA_2_s, for instance, are highly conserved between species and commonly employed as pharmacological tools to investigate their role in many clinical states [85]. A variety of deleterious effects have been observed in individuals exposed to these substances, including neurotoxicity, myotoxicity, cardiotoxicity, cytotoxicity, anticoagulation, convulsions, hypotension, and proinflammatory responses, among other manifestations. 

Numerous snake venoms have been identified as containing PLA_2_ neurotoxins, which exert their effects on neuromuscular junctions. The PLA_2_ neurotoxins encompass β-bungarotoxin, Taipotoxin, Crotoxin, and Notexin. Although neurotoxins vary in their structures and origins, their primary characteristic is the inhibition of acetylcholine release from motor nerves [86]. In this discussion, we will explore the structural and functional characteristics of Kv channel blockers.

#### 3.2.1. Crotamine

The initial discovery of crotamine occurred through the isolation process from the venom of the South American rattlesnake species known as *Crotalus durissus terrificus* [87]. This particular substance is classified within the category of short basic polypeptide myotoxins (SBPM), which are commonly found in the venoms of *Crotalus* rattlesnakes. The SBPMs exhibit variations across individuals based on factors such as subspecies or geographical location [88,89].

#### The Molecular Structure of Crotamine

**Figure 4:**
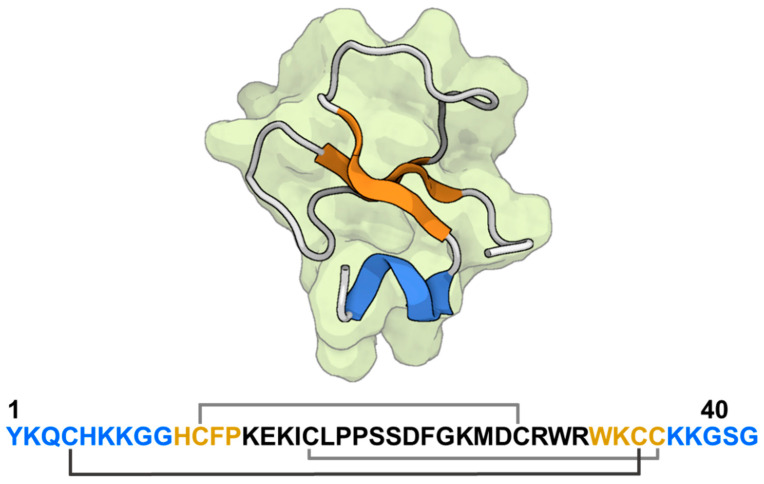
The 3D molecular structure of crotamine and its amino acid sequence. The surface model of crotamine from *Crotalus durissus terrificus* (PDB: 1H5O; [74]). The colored parts reflect the secondary conformation structures of the peptides (the blue for the α helix, orange for the β-sheets, and the loops in gray. The 42 amino acids sequence of the crotamine shows the three disulfide bridges and the sequence in colors correlated to the spatial conformations and location in the structure (adapted from [75]). The molecular structure was visualized by Biorender.com, accessed on 9 November 2023.

Crotamine is a peptide consisting of 42 amino acids, characterized by its non-enzymatic and non-cytolytic properties. It has a molecular mass of around 4.8 kDa. The 3D solution structure of crotamine was determined using proton NMR spectroscopy (Figure 4; [90,91,92]) and subsequently confirmed via X-ray crystallography [93]. The peptide has a total of 11 basic residues, including 9 lysines, and 2 arginines. These residues contribute to the peptide’s overall positive charge, resulting in a pI value of 9.54. Additionally, the peptide contains six cysteine residues that are connected through three disulfide bridges. The aforementioned studies have also demonstrated that crotamine is composed of a brief N-terminal α-helix spanning residues 1 to 7, two anti-parallel -sheets consisting of residues 9 to 13 and 34 to 38, and two -turns encompassing residues 14 to 16 and 27 to 34 [90,92]. Consequently, a proposition was put up suggesting that the toxin possessed a β1αβ2β3conformation, wherein the first and second strands exhibited an antiparallel orientation with respect to the third strand, and the β-sheet displayed a right-handed twist. The right-handed -sheet is kept stable by two hydrogen bonds between strands 2 and 3 and four hydrogen bonds between strands 1 and 3 (residues 10–37 and 12–35). Subsequently, more scholars documented the existence of a specific structural arrangement consisting of only two β-sheets, which adopt the αβ1β2 topology. This configuration has a single α-helix encompassing residues 1–7, along with two antiparallel β-sheets composed of strands spanning residues 9–13 and 34–38 [92]. The protein is stabilized by three disulfide bridges, specifically at positions C4–C36, C11–C30, and C18–C37 [90,92]. It is noteworthy that crotamine exhibits a similar disulfide bridge arrangement as members of the human β-defensin family, and it likewise features a closely aligned structural fold conformation [94]. Therefore, crotamine is classified as a peptide that has similarities to β-Defensins. The existence of two isoforms of crotamine has been identified, with their differentiation attributed to a substitution of leucine 19 by an isoleucine residue [91,95].

#### Crotamine: Interesting Molecule with Various Pharmacological Potentials

Crotamine exhibits a diverse range of pharmacological activities, such as the stimulation of acetylcholine and dopamine release in striated muscular tissue [96], the induction of histamine release in mast cells [97], analgesic effects [98], the promotion of insulin secretion in β-pancreatic cells [99], and the enhancement of macrophage phagocytic activity through the activation of nitric oxide (iNOs) and TNF-α via p38 and nFk-B pathways [100]. According to a study on animal behavior, it was shown that crotamine had a positive impact on the spatial memory retention of mice, without affecting their mood, anxiety levels, or locomotion [101]. 

Crotamine is a peptide with cell-penetrating properties that specifically targets chromosomes, facilitating the transportation of plasmid DNA into cells. Moreover, it has a notable preference for actively dividing cells [95]. Cell division involves the presence of centrosomes, which may be observed within the nucleus as they associate with chromosomes and centrioles. The inherent positive charge of this toxin enables its binding to the negatively charged DNA in a manner that lacks cooperation [102]. As a result, the process of fluorescently labeling crotamine and its subsequent attachment to the chromosome during metaphase leads to the formation of a discernible banding pattern. The antitumor efficacy of crotamine was shown in multiple aggressive tumorigenic cell lineages while exhibiting no activity against normal cells [103]. Hence, Chen et al. [102] advocated that more investigations should be conducted to explore the potential of crotamine as a proficient carrier for nucleic acid drugs, specifically targeting actively proliferating cells, such as tumor cells.

Furthermore, it has been demonstrated through in vivo experiments that crotamine may be detected in distinct animal tissues, including the liver, skeletal muscle, bone marrow, and kidney [104]. Moreover, it has demonstrated the ability to traverse the BBB, as evidenced by its presence within the brain through the utilization of fluorescently labeled and radiolabeled crotamine [105]. The process of crotamine transcytosis across the BBB can be divided into three distinct stages. Firstly, crotamine binds to and is taken up by endocytosis on the luminal side of the endothelial cell membrane, which is negatively charged due to its high content of chondroitin and heparan sulfate molecules. Secondly, crotamine diffuses through the cytoplasm of the endothelial cell. Lastly, crotamine is exocytosed from the endothelial cells [106]. In addition to its classification as the initial venom peptide with natural cell-penetrating properties, crotamine exhibits antibacterial characteristics, particularly demonstrating a heightened antifungal efficacy [107]. Additionally, it exerts its effects on bacteria via the oxidative stress pathway [108]. 

Crotamine exhibits myonecrotic action through the process of vacuolization, which leads to structural destruction of the skeletal muscle membranes [109]. Crotamine was administered to mice at sublethal doses of 2.5 mg/kg body mass, with an LD_50_ value of 33 μg/g [90]. This resulted in hind-limb paralysis and necrosis of muscle cells. Crotamine has been observed to induce cytotoxicity through its ability to disrupt cellular homeostasis, resulting in damage to many cytoplasmic organelles, including lysosomes and mitochondria [110]. Crotamine exhibits a specific affinity for the mitochondrial respiratory chain, suggesting its potential as an uncoupling agent in tandem with the creation of mitochondrial membrane permeability holes [108]. Furthermore, it has been observed that crotamine exhibits a significant pro-aggregation action on platelets. 

Crotamine induces irreversible depolarization of cellular membranes, resulting in muscle contractions through the augmentation of sodium ion permeability in skeletal muscle membranes [111,112]. Additionally, it elicits spontaneous repeated firing in mammalian skeletal muscle [113,114]. The depolarizing action of crotamine in the diaphragm muscle of mice was examined by Chang and Tseng [113]. Their study indicated that this depolarization is primarily caused by the impact on Na^+^ channels. They further observed that the depolarization could be noncompetitively reversed by substances such as tetrodotoxin (TTX), procaine, and high calcium and low sodium media [113]. The involvement of Na^+^ channels in this phenomenon has been a subject of contention among multiple investigations. According to a prominent study, it was demonstrated that the depolarization of membrane potential induced by crotamine can be prevented and restored by TTX. However, TTX does not hinder the irreversible binding of crotamine. Furthermore, the study acknowledged that the interaction site of TTX differs from that of crotamine [115]. In contrast, the study conducted by Rizzi et al. in 1998 provided evidence indicating that crotamine does not have a direct impact on the mammalian Nav1.1 to 1.6 channels that are expressed on HEK293 cells and are also naturally occurring in DRG neurons. The findings of this study were further corroborated by comparing the envenoming behavior of crotamine with those of established Nav toxins (TTX, μ-Conotoxin-GIIIa, BcIII, Tx2-6, α-pompilidotoxin, and β-pompilidotoxin), all of which failed to reverse the hind-limb paralysis induced by crotamine [114]. Subsequently, a proposition was made regarding the potential of crotamine to function as a Kv channel blocker. This proposition was based on the observation that crotamine’s three-dimensional structure has similarities to that of human antibacterial β-defensins. In order to support this proposition, in silico docking techniques were employed [116].

In accordance with Yount et al.’s computational investigation, Peigneur et al. conducted a study that provided functional evidence [106]. Their findings revealed that crotamine exhibits selective and potent blocking effects on mammalian Kv1.1, Kv1.2, and Kv1.3 channels. These channels were expressed heterologously in *Xenopus laevis* oocytes. This study encompassed a total of 16 distinct Kv channel types, including Kv1.1–Kv1.6, Kv2.1, Kv3.1, Kv4.2–4.3, Shaker IR, and hERG [106]. The collective investigation showed IC_50_ values of around 370, 400, and 300 nM for Kv1.1, Kv1.2, and Kv1.3 channels, respectively. The conducted study by Peigneur et al. proposed that the residues Tyr1 and Lys2, along with Arg31 and Trp32, could have significant functional roles in the blockage of Kv1 channels by crotamine (as depicted in Figure 4). However, no mutagenesis investigation has provided evidence supporting the assumption that these surface residues are part of a dyad pharmacophore. It is important to acknowledge that such a study provides further insight into the potential impact of Kv channel blocking on its anticancer properties. However, additional research is necessary to fully elucidate the specific mechanisms underlying this involvement [106]. The observation that crotamine exhibits activity on Kv channels could serve as a foundation for further investigations into defensin-like peptides found in snake venom and other venomous organisms, which may similarly demonstrate an affinity for Kv channels.

#### 3.2.2. β-Bungarotoxin

##### The Molecular Structure of β-Bungarotoxin

**Figure 5:**
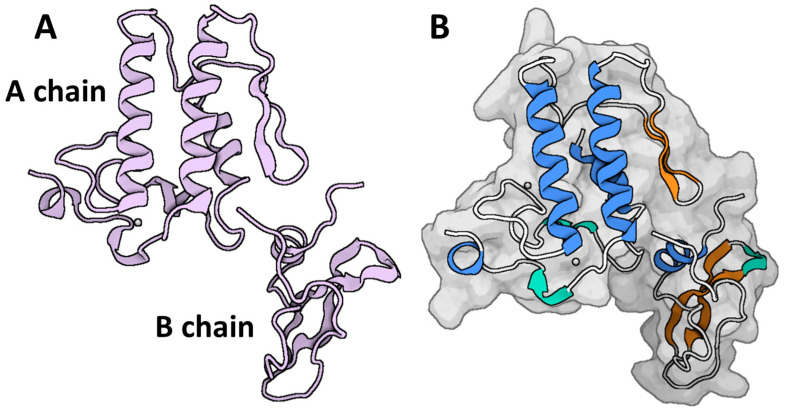
The structure of β-bungarotoxin from the Taiwan (many) banded krait *Bungarus multicinctus* (PDB: 1BUN; [121]). (**A**) The structure is composed of two chains: A and B. (**B**) The surface model of β-bungarotoxin combining the two chains. The colored parts reflect the secondary conformation structures of the peptides (the blue for the α helices, orange for the β-sheets, hinges in cyan, and the loops in gray). The molecular structure was visualized by Biorender.com, accessed on 9 November 2023.

Chang and Lee were the first to identify and conduct a pharmacological characterization of the PLA_2_, β-neurotoxin. The toxin is isolated from the venom of the Taiwan banded krait, scientifically known as *Bungarus multicinctus* (also referred to as the Formosan banded krait) [117]. The comprehensive investigation of the structure and amino acid sequence of β-bungarotoxin did not occur until 1978. β-bungarotoxin is a protein having basic properties, as shown by its pI value of 9.5. It consists of two polypeptide subunits and has a combined molecular mass of ~22 kDa [118,119]. The A-chain of the molecule exhibits homology to the PLA_2_ component, whilst the B-chain shows homology to the polypeptide of the BPTI. The connection between the two chains is facilitated by a disulfide bridge, as depicted in Figure 5. A total of sixteen isoforms of β-bungarotoxin have been successfully identified [120]. The β1-isoform, comprising around 14% of the crude venom of the Formosan banded krait, is well recognized as the predominant isoform extracted from their venoms. Kwong et al. conducted a study in which they determined the X-ray crystal structure of β_2_-bungarotoxin [121], as depicted in Figure 5. Based on the findings of this investigation, it can be observed that the structure of the B-chain exhibits notable dissimilarities when compared to the native BPTIs. These structural variations provide an explanation for its inability to operate as a protease inhibitor. Likewise, it has been observed that the binding area of the A-substrate chain is predominantly obstructed, leading to diminished enzymatic activity [122].

The A-chain is a polypeptide with a molecular mass of ~13 kDa. It is characterized by its neutral nature, with a pI value of 7.4, and it has a relatively low PLA_2_ activity. The A-chain consists of a linear sequence of 120 amino acid residues, connected by six disulfide bridges [119]. The A-chain exhibits a striking similarity to both swine pancreatic PLA_2_ and Notexin, a presynaptic neurotoxin found in the Australian elapid *Notechis scutatus*. It is well recognized as the active subunit of the β-bungarotoxin complex, possessing both neurotoxic and phospholipase activity [119,123,124]. The B-chain, referred to as the “Kunitz subunit ” [124], is the main subunit with a molecular mass of ~7.5 KDa and a basic pI of 9.6. Wu et al. [125] discovered that the B-chain consists of a sequence of 61 amino acids arranged in a linear structure connected by three disulfide bridges. The functional peptide toxin is produced by the linkage of the B-chain with the A-chain at position 55 by an additional bridge. In fact, the separation of the A-chain and B-chain components of β-bungarotoxin was achieved effectively through the targeted elimination of the interchain bridge responsible for their linkage [121]. While the fragmented chain demonstrated the ability to connect with calcium, neither of the isolated chains exhibited the same level of lethality as the intact native structure. 

##### The Pharmacology of Presynaptic Inhibition of Neurotransmission by β-Bungarotoxin

The B-chain of the β-bungarotoxin exhibits homology with snake venom protease inhibitors of the Kunitz type, as well as with DTXs [123,124]. The peptides’ convergent similarity elucidates their impact on a shared molecular target, likely the Kv1 channels located on nerve terminals [126]. The induction of continuous firing in the nerve following single shock nerve stimulation at the mouse neuromuscular junction is facilitated by DTX, which acts by inhibiting Kv1 channels and promoting neurotransmitter release [67]. As a result, the occurrence of multiple end-plate potentials and repetitive muscle action potentials subsequently leads to an increase in muscle twitch amplitude. 

In a series of trials involving several animal species, it was observed that chicks had the most susceptibility to the lethal effects of β-bungarotoxin, followed by guinea pigs and rats. Conversely, mice were found to be the least sensitive to the toxin’s lethal activity [127]. In mice, the LD_100_ of β-bungarotoxin following intraperitoneal injection is reported to be 10 mg/kg [118,128]. Respiratory failure and neuromuscular blockade are the primary causes of mortality, primarily resulting from the inhibition of acetylcholine release [129]. The precise processes responsible for the early modifications in transmitter release caused by β-bungarotoxin, as well as its subsequent inhibition of transmitter release, remained unclear. The accurate quantification of electrophysiological activity associated with transmitter release poses a significant challenge in initial investigations, mostly due to the limited dimensions of nerve terminals in skeletal neuromuscular synapses. Subsequently, experiments were conducted on the neuromuscular junction, specifically on synaptic membranes derived from brain tissue, to investigate the effects of β-bungarotoxin on neurotransmission in these tissues.

β-bungarotoxin functions by exerting a pre-synaptic effect that hinders neuromuscular transmission through the augmentation of transmitter release. Initially, the firing of action potentials is observed, which is subsequently succeeded by the complete cessation of the spontaneous small end-plate potentials [118,130]. The observed inhibition does not have an impact on the sensitivity of acetylcholine at the end-plate, and the administration of δ-tubocurarine prior to toxin exposure does not impede the blocking effect. In contrast, the activity of β-bungarotoxin results in the depletion of synaptic vesicles, whereas botulinum toxin exerts its effects at the pre-synaptic level. The adverse effects of β-bungarotoxin encompass a range of symptoms, such as headaches, dizziness, visual and speech impairments, loss of consciousness, convulsions, gastrointestinal discomfort, and muscular paralysis. These toxic manifestations can endure for a duration of up to four days, and in severe cases, respiratory paralysis can lead to fatality [118,130]. In contrast, previous studies have demonstrated that dendrotoxin inhibits the binding of β-bungarotoxin to synaptic membranes derived from the brains of rats, chicks, and guinea pigs. Additionally, it has been observed that DTX induces a delay in the neuromuscular blockade induced by β-bungarotoxin [48,131]. Based on the aforementioned data, it has been postulated that these two molecules exhibit a shared receptor. The acceptor is composed of two polypeptide chains weighing 65 and 39 kDa, respectively. The N-terminal sequence of the 65 kDa polypeptide closely matches the N-terminal sequence inferred from the cDNA of Kv1.2, which is a frequently targeted protein by dendrotoxins [57,132]. 

In previous studies, it has been observed that the presence of β-bungarotoxin in mouse motor nerve terminals leads to the inhibition of a K^+^ current. This effect has been investigated in both normal physiological salt solutions and in solutions lacking Ca^2+^. Additionally, similar results were obtained when Sr^2+^, which act as an inhibitor of PLA_2_, were used as a substitute for Ca^2+^ ions in the solutions. On the other hand, it has been shown that β-bungarotoxin does not exhibit inhibitory effects on Na^+^ currents, calcium-activated potassium currents, or calcium currents originating from motor nerve terminals in mice [133,134]. In contrast to DTXs, the inhibition of neuronal Kv channels by β-bungarotoxin does not induce recurrent nerve firing. Similar to DTX, the blockage of the K^+^ channel by β-bungarotoxin is not reliant on enzymatic activity. Therefore, it is widely agreed upon that the action of β-bungarotoxin involves the inhibition of a Kv channel. This inhibition leads to a prolongation of the repolarization phase of the nerve terminal following an action potential. Additionally, it results in the extended opening of Cav channels, leading to an increase in the concentration of Ca^2+^ within the nerve terminal. As a result, it can be believed that the increased concentration of Ca^2+^ would lead to an augmentation in the secretion of acetylcholine. The initial discovery of the inhibitory effect of β-bungarotoxin on K^+^ currents was made by Petersen et al. [135]. In the conducted studies, a concentration of 45 nM of β-bungarotoxin was seen to exhibit partial blocking effects on a non-inactivating K^+^ current in guinea pig DRGs. In addition, previous studies have shown that β-bungarotoxin has the ability to partially inhibit a DTX-sensitive K^+^ current in myelinated nerve fibers of the *Xenopus* [136]. 

Previous research has shown that the B-chain, when isolated either through chemical separation or selective expression, possesses the capacity to inhibit Kv channels [125]. Therefore, it is probable that the B-chain is accountable for the inhibition of specific Kv channels. The specific subtype(s) of Kv channels that are inhibited by β-bungarotoxin remains uncertain. Nevertheless, according to the capacity of β-bungarotoxin to displace DTX-I from its receptor, two proteins have been documented: one exhibiting a high affinity (IC_50_ = 10 nM) and another displaying a low affinity (IC_50_ = 560 nM) [49]. However, it has been established that β-bungarotoxin effectively inhibits Kv1.2 channels that are present in *Xenopus* oocytes, with an IC_50_ value of 50 nM [137]. Additionally, it has been seen that this toxin also blocks type 1 currents, which are comparable to Kv1.1 channels, in rabbit Schwann cells that have been cultivated from neonatal sciatic nerves, with a K_d_ value of 46 nM [138]. In their study, Lin et al. [139] employed yeast two-hybrid screening and pull-down assay techniques to identify KChIP3 as a binding protein for the B1-chain of β-bungarotoxin. Given the diverse roles of KChIP3 in neuronal cells, its presence may serve as a potential mechanism for the expression of the toxin’s biological effects.

#### 3.2.3. Natratoxin 

##### The Molecular Structure of Natratoxin

**Figure 6:**
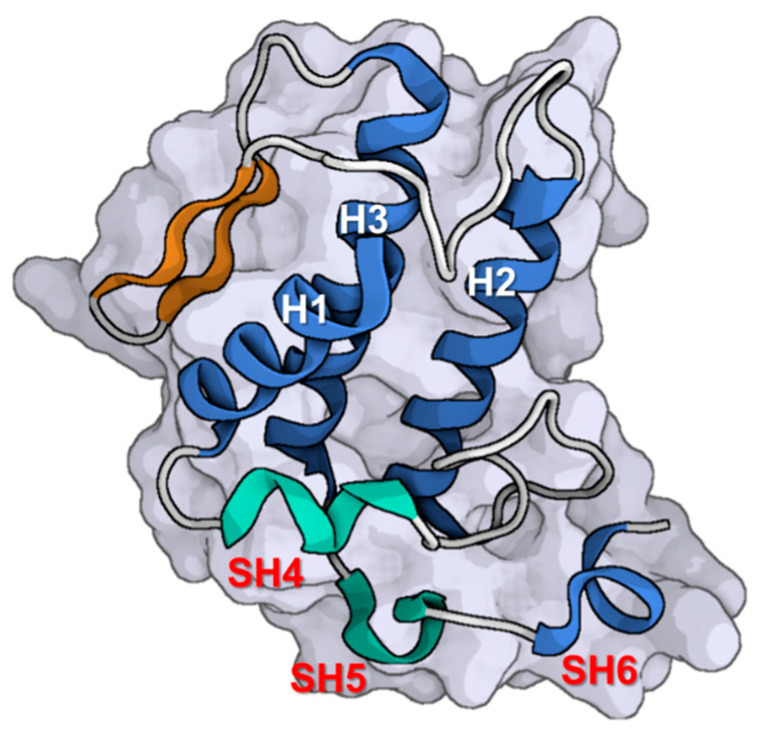
The 3D structure of natratoxin. The surface model of natratoxin from the Chinese cobra *Naja atra* (PDB: 2OSH; [140]). The colored parts reflect the secondary conformation structures of the peptides: the blue for the α helices (H1–H3), orange for the β-sheets, hinges in cyan for SH4 and SH5, the terminal SH6 α helix in blue, and the loops in gray. The molecular structure was visualized by Biorender.com, accessed on 9 November 2023.

Natratoxin is a member of the sPLA_2_s family that is released by snakes, specifically identified from the venom of *Naja atra*. The molecular structure of natratoxin exhibits all the essential properties commonly found in PLA_2_-type enzymes. The structure of natratoxin, as depicted in Figure 6, is composed of six helices and a double-stranded antiparallel β-sheet that is stabilized by seven disulfide bridges. The six helices consist of an N-terminal helix 1 (H1) spanning residues 2 to 12, helix 2 (H2) spanning residues 39 to 55, and helix 3 (H3) spanning residues 84 to 102. Additionally, there are three helical short turns including residues 19–21 (SH4), 107–109 (SH5), and 114–116 (SH6). The residues spanning from 69 to 72 and 75 to 78 exhibit a double-stranded antiparallel β-sheet structure [140].

The rationale behind the appropriate binding orientation of natratoxin is elucidated by the spatial separation between the amino acids Arg115 and Lys117 at the C-terminus of the protein. This geographical distance facilitates electrostatic interactions between the positively charged residues of natratoxin and the negatively charged residues of the Kv4 channel, hence directing natratoxin toward the optimal binding conformation [140]. Beneath the positively charged residues, Arg115 and Lys117, lies an Asp113 residue that facilitates the formation of a dipole moment by assuming a negatively charged state. The residues Ile112 and Tyr110, which are situated in the vicinity of Asp113, facilitate the establishment of hydrophobic interactions between the toxin and the channel. These interactions enable natratoxin to adopt the appropriate orientation [141]. Consequently, the collective physiochemical characteristics of its functional surface are consistent with those exhibited by currently available inhibitors of Kv channels.

##### Natrotoxin Is Targeting the Fast-Inactivation Kv Channels

The fast-inactivating K^+^ current (I_A_) in dissociated DRG neurons was found to be inhibited in a concentration-dependent manner by natratoxin. Furthermore, it exerted an influence on the kinetics of channel gating, leading to alterations in the steady-state of activation and inactivation curves in the direction of hyperpolarization, along with modifications in V_1/2_ and the slope factor [140]. Similar to neurons in other regions, it is believed that the I_A_ current in DRG neurons plays a role in regulating the timing of recurrent action potential generation, the repolarization of individual action potentials, and the duration needed to reach the threshold for firing an action potential [142,143]. The role of I_A_ is also believed to be involved in nociception in DRG neurons [144,145]. The aforementioned discoveries have the potential to enhance comprehension of the peripheral neurotoxicity associated with the sPLA_2_ neurotoxin present in snake venom, specifically in relation to the nociceptive consequences observed in cases of human intoxication from snake bites. The pharmacological characteristics of I_A_ in rat DRG neurons exhibit similarities to the Kv4 (Shal) family of K^+^ channels [146,147]. These channels are known to activate at subthreshold membrane potentials, display rapid inactivation, and exhibit efficient recovery from inactivation [148,149]. It is noteworthy to notice that natratoxin has the ability to reduce the amplitude of the I_A_ current, while also causing alterations in the kinetics of channel gating. This suggests that natratoxin may function as a modulator of channel gating. The inhibitory effect is not mediated through catalytic activity. The occurrence can also be explained by the presence of distinct binding sites on the surface, separate from the catalytic sites.

It is hypothesized that the functional region located at the C-terminus of natratoxin is involved in the interaction with specific regions of the I_A_ channel that are in close physical proximity to the S4 VSD, hence exerting an influence on the gating mechanism of the channel [140]. The process of binding to the Kv4 channel in its resting state has the potential to induce aberrant conformational changes in the channel. This, in turn, can impede the passage of K^+^ ions and enhance the voltage sensor’s responsiveness to depolarization of the membrane potential. Consequently, the peak amplitude of I_A_ was diminished, and the activation curves exhibited a negative shift. Furthermore, the magnitude of the voltage alteration observed in the channel inactivation was greater than that observed in the channel activation. The activation and inactivation of the Kv channel are regulated by distinct processes. The utilization of an N-type inactivation domain is a prevalent strategy employed by swiftly inactivating Kv channels of the A-type [140]. In N-type inactivation [150], the presence of a tethered N-terminal inactivation domain obstructs the pore subsequent to channel opening. This obstruction effectively sustains the channel in an open-inactivated state, preventing its closure [151]. Nevertheless, the inactivation mechanism observed in I_A_ is not applicable to the Kv4 channels, which belong to the *Shal* family. Following a brief period of occupying an open-inactivated state, Kv4 channels undergo aggregation into a closed-inactivated state (Ic) when subjected to sustained depolarization (I_o_) [152]. The binding process induces a conformational change in the channel, resulting in its conversion to a closed inactivated state that is more stable. Consequently, the voltage shift associated with channel inactivation may exhibit a greater magnitude. Further investigation into the relationship between structure and function is necessary in order to have a more comprehensive understanding of the binding mechanism in question.

#### 3.2.4. MiDCA1

Belo et al. (2005) [153] were the first to successfully isolate a polypeptide, known as MiDCA1, from the venom of the coral snake *Micrurus dumerilii carinicauda*. This polypeptide has a molecular mass of ~15.5 kDa [153]. The toxin has a significant degree of sequence homology with the PLA_2_ toxins found in the venom of *Micrurus nigrocinctus*. In experiments involving chick biventer cervicis preparations, the administration of MiDCA1 resulted in a neuromuscular blockade that was dependent on both the concentration and duration of exposure. The blockade achieved its maximum effect of 100% following a 2 h incubation period at a concentration of 2.4 μM. The application of an equivalent quantity of MiDCA1 elicited triphasic alterations, which is succeeded by partial inhibition of neuromuscular activity in preparations of a mouse’s phrenic nerve–diaphragm. The quantal content of end-plate potentials (EPPs) and miniature end-plate potentials (MEPPs) in mouse diaphragm preparations was measured using intracellular recordings. The application of MiDCA1 resulted in a nearly four-fold increase in the quantal content after 10 min. Additionally, the frequency of MEPPs exhibited a triphasic variation in response to MiDCA1 [153]. The resting membrane potential was found to be reduced by MiDCA1. This effect was seen to be blocked by tetrodotoxin and/or low extracellular calcium, but not by δ-tubocurarine. In addition, the toxin elicited an augmentation in the amplitude of compound action potentials in the sciatic nerves of mice. In the aforementioned investigation, the K^+^ currents observed in newly dissociated DRG neurons were shown to be inhibited by approximately 30% when exposed to a concentration of 2.4 μM of MiDCA1. The lack of efficacy of δ-Turocurarine in inhibiting the depolarization caused by MiDCA1 suggests that cholinergic nicotinic receptors do not play a role in this phenomenon. In a distinct study, it was established that the polypeptide consisting of 120 amino acids exhibited diminished enzymatic activity and displayed moderately basic characteristics, as evidenced by its pI of 8.0 [154]. MiDCA1 was identified as a Kv channel antagonist through several electrophysiological investigations. Initially, the toxin demonstrated inhibitory effects on a prominent element of Kv currents within cultured DRG neurons of mice [155]. In the context of cultured neurons, it was observed that MiDCA1 exhibited competitive inhibition of the outward K^+^ current, in comparison to Guangxitoxin, a well-established selective blocker of the Kv2.1 channel [155]. In a series of tests utilizing the *Xenopus* oocyte heterologous system, it was observed that the application of MiDCA1 at a concentration of 1 µM resulted in a reversible inhibition of the Kv2.1 current, reducing it by around 60% [155]. The toxin exhibited a notable preference for Kv2.1 channels compared to the other Kv channels examined in the aforementioned study; namely, Kv1.1-1.6 and KCNQ1-2 [155]. These results explain how the toxin affects the release of acetylcholine at mammalian neuromuscular junctions. 

#### 3.2.5. Taipoxin, Notexin, and Crotoxin

**Figure 7:**
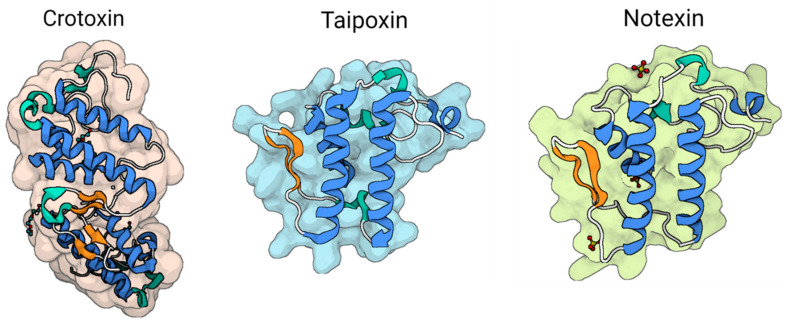
The 3D molecular structures of some PLA_2_ toxins: crotoxin (PDB: 3R0L; [158]), taipoxin (PDB: 3VC0; [143]), and notexin (PDB: 1AE7; [144]). The colored parts reflect the secondary conformation structures of the peptides: the blue for the α helices, orange for the β-sheets, hinges in cyan, and the loops in gray. The molecular structure was visualized by Biorender.com, accessed on 9 November 2023.

Taipoxin, often known as taipan toxin, is the primary myo- and neurotoxic constituent, in conjunction with notexin, derived from the venom of the Australian taipan (*Oxyuranus s. scutellatus*) (Figure 7, [156,157]). The notable characteristic of the toxicology of these molecules is the degradation of nerve terminals and axons. The injection of taipoxin and notexin into the hind limb of rats resulted in the rapid depletion of transmission from the motor nerve terminals of the soleus muscle within one hour. Subsequently, there occurred the deterioration of the motor nerve terminals and the axonal cytoskeleton. Within a 24 h period, a significant proportion of muscle fibers, specifically 70%, underwent full denervation. The complete recovery of this impact was observed after a duration of five days [157].

The lethal dose 50 (LD_50_) of taipoxin in mice is reported to be 2 μg/kg. This toxin primarily acts by gradually inhibiting the release of acetylcholine from motor nerve terminals, resulting in a total cessation of both evoked and spontaneous acetylcholine release. Ultimately, this physiological impact leads to suffocation. Taipoxin is a sialo-glycoprotein of moderate acidity, with a calculated pI value ~5. It has a molecular mass of approximately 4.6 kDa and consists of three subunits; namely, alpha, beta, and gamma subunits. These subunits are interconnected by the formation of seven disulfide bridges, as reported in the literature [143]. The β component, which has a pI value below 10, consists of 13 arginine residues and is the sole subunit that exhibits deadly neurotoxicity, with a mouse LD_50_ value of approximately 300 μg/kg. Notexin is a polypeptide consisting of 119 amino acid residues that are connected by seven disulfide bridges (as shown in Figure 7; Ref. [144]). Notexin, similar to taipoxin, causes rapid death by inducing asphyxiation. This is accomplished by inhibiting signal transmission across the neuromuscular connections that control the breathing muscles. 

Crotoxin, a presynaptic polypeptide, was the first compound to be extracted from *Crotalus durissus terrificus* snake venom [158,159,160,161]. The toxin exhibits presynaptic neuromuscular blocking, a triphasic effect on neurotransmitter release, as well as myotoxicity, cardiotoxicity, and nephrotoxicity, as discussed in a previous review [161]. Crotoxin is composed of two distinct components: a basic, moderately toxic PLA_2_ and an acidic, non-toxic, non-enzymatic component known as crotapotin. This heterodimeric complex is depicted in Figure 7 [158]. 

The PLA_2_ toxins, including β-bungarotoxins, taipoxin, notexin, and crotoxin, were found to exhibit a comparable ability to impede the neuromuscular transmission in mice by suppressing the terminal K^+^ component. This action was observed to be independent of phospholipase activity [134]. The present study aimed to evaluate the effects of dendrotoxin, β-bungarotoxin, crotoxin, taipoxin, bee venom PLA_2_, aprotinin, and apamin on presynaptic currents in mouse triangularis sterni [133]. The observed neurotoxins did not exhibit inhibitory effects on the I_A_ or the IK_Ca_ channels. However, they did demonstrate a significant and irreversible blockade of the presynaptic slowly activating K^+^ currents. The data presented suggest that the facilitatory effects of dendrotoxin, β-bungarotoxin, taipoxin, and crotoxin are brought about by an elevation in the influx of calcium ions into nerve terminals. Remarkably, these toxins exhibited no inhibitory effect on whole-cell patch clamp recordings obtained from mammalian cell lines that were stably expressing mKv1.1, rKv1.2, mKv1.3, hKv1.5, and mKv3.1 [86]. Taken together, these findings indicate that the manner in which these neurotoxins enhance the release of acetylcholine at the neuromuscular junction is not associated with the direct inhibition of these channels. Additional research should be conducted in order to determine the molecular target of these toxins.

### 3.3. Cysteine-Rich Secretory Proteins (CRISPs)

Cysteine-rich secretory proteins (CRISPs) are a widely distributed collection of polypeptides found in snake venoms. The protein under investigation has a molecular mass range of approximately 20 to 30 kDa. It is composed of approximately 220 amino acid residues and possesses a particular pattern of 16 cysteine residues that are highly conserved. Each member of the CRISP family is characterized by the presence of 16 cysteine residues that are tightly conserved, and these cysteines are responsible for the formation of eight disulfide linkages. Certain members of the CRISP family have the ability to inhibit L-type Ca^2+^ channels, cyclic nucleotide-gated ion channels, and BK_ca_ channels [162,163,164].

#### 3.3.1. BaltCRP 

In their study, Bernardes et al. [165] conducted the isolation and characterization of a CRISP (cysteine-rich secretory protein) from the venom of *Bothrops alternatus*, which they dubbed BaltCRP. They then investigated the impact of BaltCRP on several isoforms of K^+^ channels in vitro, as well as its impacts on inflammatory processes in vivo. The protein under investigation has a molecular mass of around 24 kDa and a theoretical pI ~7.8. The electrophysiological tests show that BaltCRP effectively inhibits the currents of Kv1.1, Kv1.3, Kv2.1, and Shaker channels within the micromolar concentration range. However, no discernible impact was observed on Kv1.2, Kv1.4, Kv1.5, and Kv10.1 channels [165]. Furthermore, the administration of BaltCRP resulted in the activation of inflammatory processes, as evidenced by an observed elevation in the number of leukocytes present in the peritoneal cavity of mice [165]. The findings of this study provide evidence that BaltCRP can contribute to the comprehension of the physiological impacts exerted by snake venom CRISPs. Enhancing our comprehension of envenomation will facilitate the advancement of more effective therapy approaches for snake bites caused by *B. alternatus*, as well as the discovery of novel therapeutic compounds.

#### 3.3.2. Natrin 

**Figure 8:**
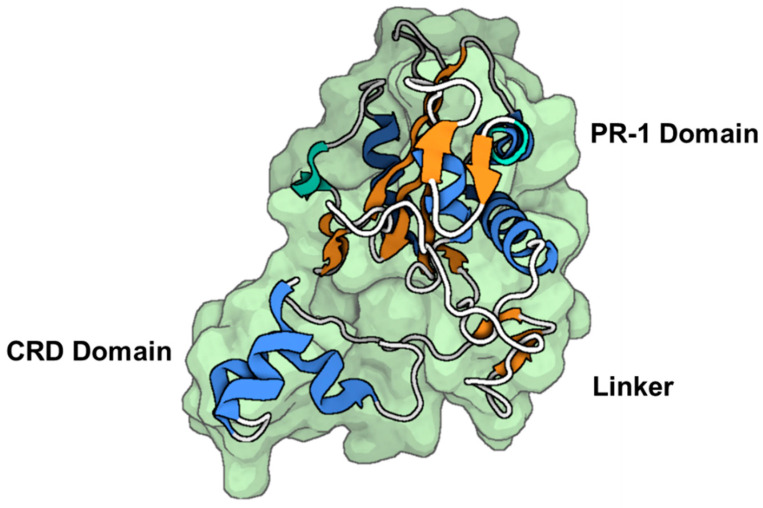
The 3D molecular structure of natrin. The surface model of natrin from *Naja atra* venom (PDB: 2GIZ; [163]). The colored parts reflect the secondary conformation structures of the peptides (the blue for the α helix, the orange for the β-sheets, the cyans for hinges, and the loops in gray). The structure is made of two parts separated by a linker PRI-1 and CRD domains. The molecular structure was visualized by Biorender.com, accessed on 9 November 2023.

The initial isolation of natrin was the extraction of the compound from the venom of *Naja atra*. This purified form of natrin was then tested for its ability to induce a contractile response in the thoracic aorta of mice, specifically in the endothelium-denuded region. Prior to this testing, the thoracic aorta had been contracted using a high-K^+^ solution [163]. The protein is composed of 221 amino acid residues, which are organized into two distinct domains. The crystallographic arrangement of a peptide-toxin with a molecular mass of ~25 kDa reveals the presence of two consecutive domains: an N-terminal domain belonging to the group 1 pathogenesis-related protein (PR-1) and a C-terminal domain rich in cysteine residues (CRD, as shown in Figure 8). 

The protein region known as the N-terminal α/β/α-sandwich motif, often referred to as the PR-1 domain, encompasses residues 1–160 [163,164]. Additionally, the C-terminal cysteine-rich domain, abbreviated as CRD, spans residues 183–221 [163]. The two domains are connected by a tight hinge region spanning residues 161 to 182. It is noteworthy that CRD exhibits structural similarities with two K^+^ channel blockers; namely, ShK from *Stichodacryla helianthus* and BgK from *Bunodosoma granulifera* [164]. This observation implies a potential relationship between this domain and the interaction with Kv channels. The polypeptide under investigation exhibited concentration-dependent inhibition of the high-conductance BK_Ca_ channel in experiments, as demonstrated by Wang et al. [163]. The IC_50_ was determined to be 34.4 nM. Natrin can inhibit BK_Ca_ channels in a concentration-dependent manner, as evidenced by its IC_50_ value of 34.4 nmol/L [163]. The CRD domain, which exhibits flexibility, is believed to have a significant impact on channel-blocking mechanisms. Additional electrophysiological tests have provided evidence that natrin inhibits the activity of Kv1.3 channels [164]. The docking investigations conducted on the interaction between natrin and Kv1.3 have identified key residues that exhibit a unique interaction pattern. This pattern differs from the two previously documented models of Kv channel inhibition known as the “functional dyad” and “basic ring” [20,164]. The recent discoveries will enhance the ability to conduct more research aimed at investigating the inhibitory interaction of natrin with different ion channels.

#### 3.3.3. Stecrisp

**Figure 9:**
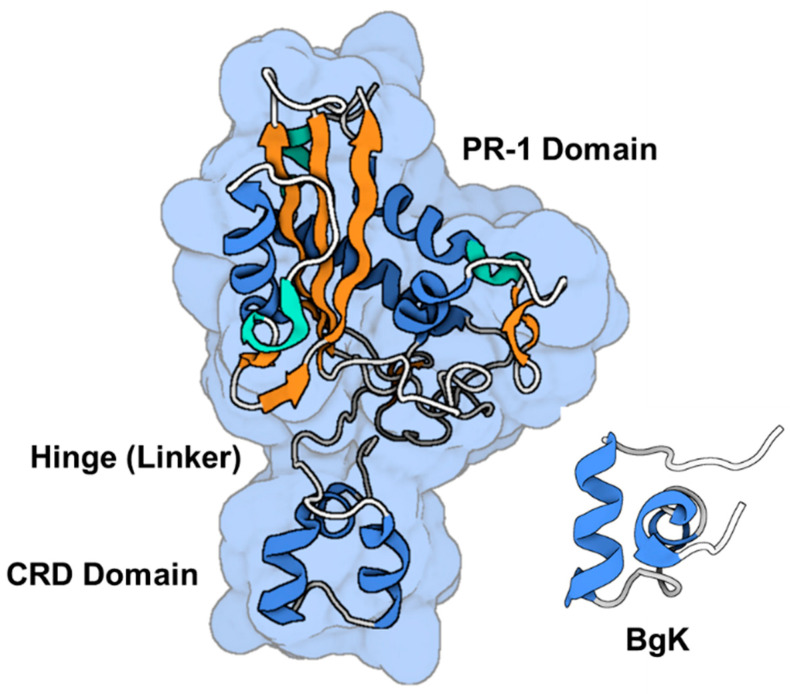
The 3D molecular structure of stecrisp. Stecrisp is first isolated from *Trimeresurus stejnegeri* snake venom (PDB: 1RC9; [166]). For comparison to the CRD domain, the Kv channel blocker from the sea anemone *Bunodosoma granulifera* (PDB: 1BGK; [141]) is presented. The colored parts reflect the secondary conformation structures of the peptides: the blue for the α helices, orange for the β-sheets, the hinges in cyan, and the loops in gray. The molecular structure was visualized by Biorender.com, accessed on 9 November 2023.

The polypeptide known as stecrisp was obtained from the venom of the *Trimeresurus stejnegeri* snake by isolation techniques conducted by Guo et al. [166]. The protein in question is a member of the CRISP family, which encompasses a range of functions including involvement in sperm-egg fusion, inherent host defense mechanisms, and the regulation of ion channels [166,167]. The crystallographic arrangement of stecrisp exhibits three discernible domains; namely, a PR-1 domain, a hinge region serving as a linker, and a CRD (Figure 9) [166,167]. The PR-1 domain has similarities with other members of the CRISPs protein family [167]. The PR-1 domain exhibits a distinctive α-β-α sandwich fold, suggesting the potential for a shared chemical mechanism. A fissure containing two histidines and two glutamate residues that remain unchanged throughout time has been observed, indicating its potential role as an active site [166,168]. In another finding, it was observed that a specific protein called Tex31, derived from the cone snail species, possesses endopeptidase activity [169]. This finding provides additional evidence supporting the notion that the PR-1 domain may serve as an enzyme module. The hinge region is stabilized through the establishment of two disulfide bonds, which are created by four out of the ten cysteine residues that are typically present in the carboxyl-terminal area. The CRD exhibits a comparable structural conformation to two Kv channel inhibitors derived from sea anemones; namely, Bgk and Shk [141]. Nevertheless, there has been a lack of research investigating the specific molecular target of stecrisp within Kv channels.

### 3.4. Snake Venom Serine Proteases (SVSPs)

Snake venom serine proteases (SVSPs) are commonly present in the venoms of snakes belonging to the Viperidae and Crotalidae families [170]. Serine proteases exert their primary effects on plasma proteins, resulting in a diverse range of physiological responses including platelet aggregation, blood coagulation, fibrinolysis, and blood pressure regulation, as well as modulation of the complement and immune systems [171,172,173].

#### Collinein-1, Gyroxin_B1.3, and BjSP 

Boldrini-França et al. [174] documented the discovery of a specific component of snake venom serine proteases (SVSPs) known as collinein-1. This particular SVSP was isolated from the venom of *Crotalus durissus collilineatus*. Collinein-1, a thrombin-like serine protease isoform with a molecular mass of 29.5 kDa, functions as an antagonist on the Kv channel hEAG1 (Kv10.1), which is relevant in cancer [175]. It also exhibits a lesser blocking effect on the hERG1 (Kv11.1) channels [175]. However, it does not have any discernible effect on the A-type current produced by the Kv1.4 channel, as well as on the delayed rectifier channels Shaker and Kv2.1 [175]. Out of the 12 voltage-gated ion channels that were examined, it was shown that collinein-1 exhibited selective inhibition of hEAG1 currents [175]. Notably, this inhibition occurred through a mechanism that was found to be unrelated to its enzymatic activity. The researchers provided evidence to support the claim that collinein-1 has a detrimental effect on the viability of the MCF7 human breast cancer cell line, which is characterized by its high expression of hEAG1 [175]. However, the researchers observed no significant impact of collinein-1 on liver carcinoma and non-tumorigenic epithelial breast cell lines (HepG2 and MCF10A, respectively), which exhibit low levels of hEAG1 expression. A computational molecular docking model was created, wherein it was suggested that the Arg79 residue of collinein-1 interacts directly with the K^+^ selectivity filter of the human Ether-à-go-go 1 (hEAG1) channel. The findings suggest that collinein-1 exhibits promise as a pharmacological agent for reducing fibrinogen levels, hence mitigating the risk of thrombus formation in specific pathological conditions and medical interventions. Additionally, collinein-1 may have utility in certain diagnostic tests and serve as a valuable tool for investigating aspects of hemostasis.

In the same investigation conducted by Boldrini-França et al. [175], it was observed that gyroxin_B1.3, a serine protease from the venom of *Crotalus durissus terrificus* [176], and BjSP, another serine protease from the venom of *Bothrops jararaca* [177], exhibited amino acid sequence similarities of 99% and 61% with collinein-1, respectively. According to the proposed docking model [175], it has been observed that gyroxin_B1.3 exhibits complete overlap with all 20 residues found in collinein-1 that interact with hEAG1. On the other hand, BjSP demonstrates a shared presence of seven conserved residues. In terms of pharmacology, it was shown that gyroxin_B1.3 had an inhibitory effect of around 60% on the hEAG1 current when administered at a concentration of 5 µM. Conversely, BjSP demonstrated a lower inhibitory effect of approximately 9% under the same conditions [175].

### 3.5. Three-Finger Toxins

#### Cardiotoxin-I

**Figure 10:**
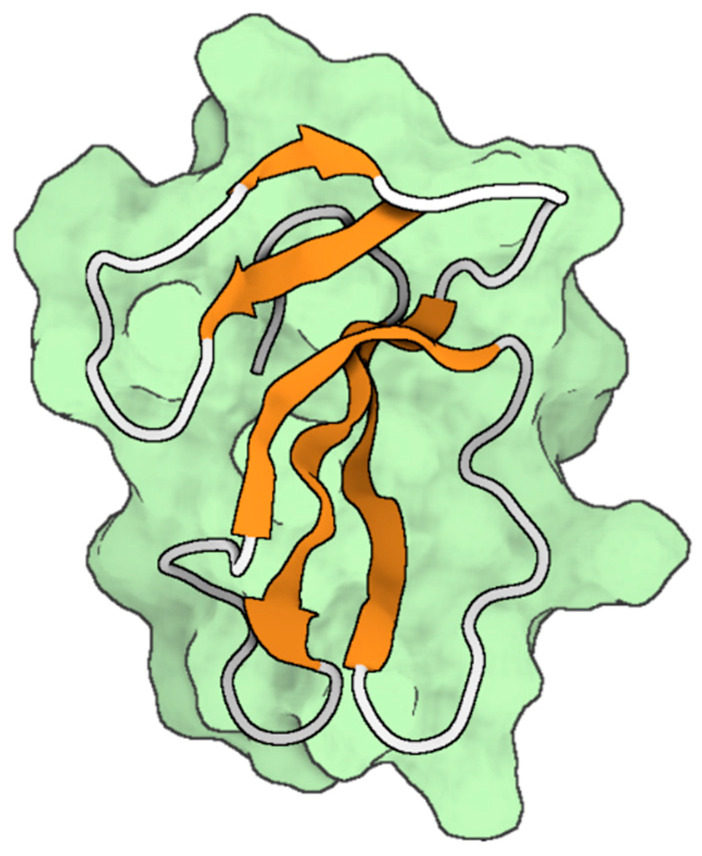
The 3D molecular structure of cardiotoxin-I. Cardiotoxin is first isolated from *Naja kaouthia* cobra snake venom (PDB: 2CDX; [179]). The colored parts reflect the secondary conformation structures of the peptides (the orange for the β-sheets, and the loops in gray). The molecular structure was visualized by Biorender.com, accessed on 9 November 2023.

Cardiotoxins (I–V) refer to a group of tiny proteins known as membrane-active proteins, which belong to the three-finger toxin family [178]. The composition of these entities consists of around 60 amino acid residues, which are stabilized through the presence of four disulfide bridges. The protein has a high abundance of beta structures, characterized by a substantial triple-stranded, antiparallel beta-sheet, together with a smaller double-stranded, antiparallel beta-sheet ([179]; see Figure 10). Cobra venom contains a significant quantity of cardiotoxins, which have demonstrated dose and time-dependent anticancer and antiproliferative effects [180]. It has been established that cardiotoxins demonstrate cytotoxic effects through both necrotic and apoptotic mechanisms [181].

ATP-sensitive potassium (K_ATP_) channels play diverse roles in different tissues, such as detecting metabolic alterations in pancreatic β-cells. This enables the connection between metabolism, electrical activity, and the eventual release of insulin [182]. Indeed, an augmentation in glucose metabolism elicits the secretion of insulin due to a raised ratio of intracellular [ATP]/[ADP], resulting in the closure of K_ATP_ channels and subsequent membrane depolarization. On the other hand, it is anticipated that a reduction in the metabolic signal will result in the activation of K_ATP_ channels, inhibiting the electrical stimulus for insulin release. Hence, the inhibition of K_ATP_ channels, which is facilitated by glucose metabolism, is frequently seen as an initial stage in the secretion of insulin [182].

The insulinotropic metabolite component in *Naja kaouthia* snake venom, specifically cardiotoxin-I, was studied by Nguyen et al. (Figure 10). In the aforementioned investigation, it was observed that the administration of cardiotoxin-I resulted in the stimulation of insulin production from a rat β-cell line, while simultaneously having no detrimental effects on the viability and structural integrity of the cells [183]. It is noteworthy that cardiotoxin-I demonstrated the ability to induce insulin production even in the absence of glucose. While cardiotoxins have been identified as powerful agents that cause hemolysis and vasoconstriction, it was shown that cardiotoxin-I did not have the ability to directly generate hemolysis in human erythrocytes or induce significant vasoconstriction. The discovery of these findings is expected to enhance the repertoire of available methodologies for investigating the physiological aspects of β-cells, and maybe introduce novel therapeutic possibilities for the treatment of type 2 diabetes.

### 3.6. Crotalphine: A Peptide Agonist for Pain Management

The management of pain related to cancer and other chronic illnesses remains a significant therapeutic concern, as the present analgesic medication has not yet yielded satisfactory outcomes. The study involved an examination of various natural sources known for their high analgesic properties, such as snake venom. The present study involved the characterization of crotalphine, a very effective analgesic peptide consisting of 14 amino acid residues. This peptide was isolated from the venom of *Crotalus durissus terrificus*, a species of rattlesnake native to South America [184,185]. The present study aimed to assess the antinociceptive properties of crotalphine in a rat model of cancer-induced pain. The induction of hyperalgesia and allodynia was shown in the animal model with the intraplantar injection of tumor cells, specifically the Walker 256 carcinoma. The administration of crotalphine at a dosage of 6 μg/kg orally effectively inhibited both occurrences within a time frame of 1 h post-treatment, with the effects persisting for a duration of up to 2 days [185]. This work involved carrying out additional pharmacological studies, which yielded results suggesting that crotalphine exhibits a robust and enduring opioid-mediated antinociceptive effect in the context of cancer pain. Moreover, the extended administration of crotalphine does not induce the formation of peripheral tolerance or withdrawal symptoms upon cessation. Upon oral administration, crotalphine elicits an antinociceptive response in rats subjected to prostaglandin E2- and carrageenan-induced mechanical hyperalgesia models, as well as in mice undergoing the hot-plate test. Crotalphine exhibits a prolonged (5 day) antinociceptive effect in mechanical hyperalgesia models [184]. The antinociceptive impact of crotalphine is facilitated through the activation of κ- and δ-opioid receptors, which occurs as a result of the activation of the NO–cGMP pathway. Additionally, this effect is mediated through the opening of K_ATP_ at peripheral afferent neurons [186,187].

There is a body of research that has addressed the involvement of various specific types of K^+^ channels in antinociception [188,189]. In addition to K_ATP_ channels, the Kir family of channels has been identified as potential targets for pain management through the use of agonist drugs in both the CNS and PNS. Specifically, these drugs aim to modulate the activity of G-protein-regulated inwardly rectifying K^+^ channels (GIRK or Kir3), Kv1.1 channels, and K_Ca_ channels (SK and BK channels), as well as the two-pore domain potassium (K_2P_) channel TREK-2 (K_2P_10.1) using specific agonists. Furthermore, it has been demonstrated that agonist medications that directly interact with neuronal Kv7 and K_ATP_ channels exhibit antinociceptive effects in both acute and chronic pain models. The findings of this study suggest that neuronal Kv channels have the potential to be a promising target for the advancement of novel K^+^ channel openers that can provide antinociceptive effects.

## 4. Snake Venom Neurotoxins as Therapeutics 

Snake venom is composed of a diverse combination of proteins and peptides, several of which have demonstrated promising medicinal possibilities [6,8,10,11,13,190,191]. The potential therapeutic applications of snake venom neurotoxins have been investigated owing to their capacity to selectively bind to certain receptors within the nervous system [192]. Neurotoxins possess the potential for both therapeutic and deleterious effects, contingent upon their distinct method of action and administered dosage. Nonetheless, the process of generating therapeutic treatments using neurotoxins derived from snake venom has numerous hurdles. One of the primary obstacles encountered in the development of snake venom neurotoxins as therapeutic agents lies in the attainment of adequate quantities suitable for clinical application [193]. The difficulties in acquiring adequate quantities of purified toxins from crude snake venom for scientific investigation and therapeutics can be mitigated by implementing venomics technologies, such as reverse-phase high-performance liquid chromatography (RP-HPLC) and followed by LC-MS/MS-based toxin identification [8]. Moreover, the future of advancements and discoveries lies in the use of efficient biotechnologies, such as cloning and large-scale toxin expression systems, and the optimization of drug delivery by toxin conjugation to monoclonal antibodies and nanoparticles [6,12]. Other approaches could involve the rational design of modified venom toxins with reduced toxicity and increased protection against proteolytic degradation [6,12]. 

A further obstacle lies in the imperative task of guaranteeing the safety of these toxins, given their high potency and the inherent risk of injury if mishandled [194]. In spite of the aforementioned hurdles, certain neurotoxins derived from snake venom have already been transformed into medicinal agents [9]. The utilization of snake venom neurotoxins exhibits considerable potential as a medicinal intervention for pain management. For instance, the antihypertensive drug Captopril (Capoten) was the first peptide derived from the venom of the *Bothrops jararaca* snake to receive FDA approval in 1981 [12,195]. This was then followed by the approval of Enalapril by the FDA in 1985 [9]. Both drugs are generated from bradykinin-potentiating peptides and function as angiotensin-converting enzyme inhibitors. They are administered to regulate hypertension and to avoid or ameliorate congestive heart failure [8,9]. This paved the way for more drug discoveries, such as Tirofiban and Eptifibatide, the selective competitive inhibitors for fibrinogen receptors [10]. 

Certain neurotoxins contained in snake venom have been discovered to specifically inhibit particular types of ion channels in sensory neurons, effectively impeding the transmission of pain signals to the brain [196]. These ion channels play a crucial role in a diverse range of pain syndromes, encompassing neuropathic pain, inflammatory pain, and cancer pain. In addition to the neurotoxins discussed in this review from various protein families (Table 2), DTXs exhibited notable efficacy and specificity against Kv1 channels. Such criteria of DTX make them useful tools to study the presynaptic Kv1 channel populations in healthy tissue and the integrity of brain’s connectomes in neurodegenerative diseases [7]. The synthesis of selective ligands against Kv1 channels, which are commonly seen in demyelinated neurons, has been achieved by rational design using a chemo-informatic approach. This strategy involves the design of chemical analogs to increase neural conduction in these neurons [197]. Nevertheless, there have been no reports of any pharmaceutical medication derived from a neurotoxic found in snake venom that specifically targets Kv channels for therapeutic purposes. Notably, Kv channel modulators derived from snake venom of various sizes (Table 2) show promise for biological uses. Due to their ability to precisely modulate various Kv channels, they have considerable potential as cardiovascular and neurological disease treatments and research tools. More research is needed to identify the molecular targets for several toxins, such as the members of PLA_2_, CRISPs, and three-finger toxins families.

Numerous toxins and derivatives resulting from the venom of snakes and other animals have demonstrated efficacy in the treatment of various medical ailments. The presence of α-cobrotoxin, a neurotoxic protein, has been identified in the venom of *Naja naja atra*, commonly referred to as the Chinese cobra [196]. This particular substance is classified as a postsynaptic neurotoxic that effectively inhibits the activity of acetylcholine at the neuromuscular junction. Shi et al. [198] examined the pain-relieving properties of α-cobrotoxin in a rat model of formalin-induced inflammatory pain. The study showed that injecting α-cobrotoxin into rats through the intraperitoneal route could reduce the inflammatory pain in a dose-dependent manner. This effect is achieved by activating the cholinergic rather than the opioid system. The beneficial antinociceptive effect of α-cobrotoxin is achieved through the activation of muscarinic and α7-necotinic acetylcholine receptors. This is in contrast to atropine, a common anticholinergic drug that acts as a nonselective antagonist for both central and peripheral muscarinic acetylcholine receptors. The drug (cobratide), derived from α-cobrotoxin, has been granted approval in China for its utilization as an analgesic in cases of moderate to severe pain [199]. Nevertheless, the considerable bioactivity of the substance may give rise to potential adverse consequences, including respiratory arrest. However, ziconotide, a pharmaceutical compound derived from the venom of the cone snail, has been authorized by both the FDA and the EMA for the purpose of managing severe chronic pain [200]. Ziconotide is a synthetic peptide that is synthesized based on the venom of the cone snail species *Conus magus* [200]. This substance serves as a non-opioid analgesic and is employed in the treatment of severe chronic pain among those who exhibit inadequate response to alternative therapeutic interventions. Ziconotide functions through the inhibition of N-type calcium channels located in the spinal cord, which play a crucial role in the transmission of pain signals [201]. Research has demonstrated the efficacy of this intervention in mitigating pain among individuals with severe and chronic conditions, such as neuropathic pain, cancer pain, and complicated regional pain syndrome [200]. In addition to the management of pain, there is ongoing research on various neurotoxins found in snake venom for its potential therapeutic applications, including the treatment of epilepsy, stroke, and hypertension [202,203]. Certain neurotoxins have demonstrated the ability to regulate the functioning of ion channels within the brain. These ion channels play a crucial role in the initiation and transmission of neuronal activity. An instance of α-neurotoxin can be observed within the venom of some snake species, such as the green mamba (*Dendroaspis angusticeps*). The α7 nicotinic acetylcholine receptor, which plays a role in modulating neuronal excitability in the brain, is specifically targeted by this neurotoxic [204,205]. The α-neurotoxin has the ability to attach to a specific receptor and inhibit its function, so mitigating the hyperexcitability that is causally linked to the occurrence of seizures. The efficacy of α-neurotoxin in mitigating seizures in rats with experimentally produced temporal lobe epilepsy has been demonstrated in preclinical investigations. Furthermore, the observed anti-seizure effects have exhibited a prolonged duration, persisting for a period of up to three days subsequent to treatment.

In addition to its Kv-blocking properties, taipoxin has demonstrated the ability to inhibit Nav channels inside the cerebral region, which play a crucial role in the initiation and transmission of neuronal action potentials. Through the process of channel blockade, taipoxin has the capacity to diminish the excitability of neurons, hence impeding the occurrence of aberrant neuronal activity that precipitates seizures. The presence of natriuretic peptide (NP), a neurotoxin, has been detected in the venom of *Calloselasma rhodostoma*, a pit viper species native to Southeast Asia. Studies have demonstrated that NP exhibits vasodilatory properties, leading to the relaxation of blood vessels and enhancement of blood circulation [191,203,206]. The relaxation of smooth muscle cells in the blood arteries is facilitated through the activation of cyclic guanosine monophosphate (cGMP) synthesis. Another illustration is contortrostatin, a neurotoxin included in the venom of the southern copperhead snake (*Agkistrodon contortrix contortrix*). The anti-angiogenic characteristics of contortrostatin have been demonstrated through its ability to suppress neovascularization [207,208]. The significance of this finding lies in its implications for the management of hypertension, as neovascularization has been identified as a potential factor in the pathogenesis of elevated blood pressure. In preclinical investigations, the administration of NP and contortrostatin have shown a notable capacity to effectively diminish blood pressure levels in rats that were experimentally produced with hypertension. Several studies have indicated that some constituents present in snake venom, such as neurotoxins, exhibit promising characteristics as possible anti-cancer agents [75,180,209,210]. The neurotoxins have demonstrated the capacity to trigger apoptosis in cancer cells and may hinder angiogenesis, the process of blood vessel formation that sustains tumor growth.

## 5. Conclusions

Gaining a comprehensive understanding of the potential of snake venom in facilitating the examination and manipulation of Kv channels represents an initial stride towards recognizing the vast yet untapped reservoir it presents. Numerous peptides and polypeptides have been extracted and examined from diverse venomous snake species. These proteins are members of families characterized by their ability to selectively and effectively inhibit and modulate important Kv channels, which are implicated in several medical illnesses including chronic pain, type 2 diabetes, cancer, and neurodegenerative diseases. The ongoing investigation and advancement of scientific inquiry are crucial in elucidating the intricate molecular mechanisms behind the actions of these neurotoxins and their potential negative consequences. The utilization of carefully engineered therapeutic agents derived from these toxins, in conjunction with advanced drug delivery methodologies, will serve as our strategy to confront forthcoming medical obstacles.

## Figures and Tables

**Table 1:** Sequences of dendrotoxin subfamilies, and a homologous protease inhibitor. pE = pyroglutamate. Taken from [50,56]. The Cys residues are in black shades and identical sequences are shaded in gray.

Toxin	Source	Amino Acid Sequence Alignment	Target Channel (s)	References
BPTI	*Bovine Pancreas*	RPDFCLEPPYTGPCKARI IRYFYNAKAGI CQTFVYGGCRAKRNNFKSAED CMRTCGGA	--	[46]
αDTX	*D. angusticeps*	pEPRRKLCILHRDPGRCYDKIPAPYYNQKKKQCERFDWESGCGGNSNRFKTIEECRRTCIG	Kv1.1, 1.2 1.6	[57,58]
DTX-I	*D. polylepis*	pEPIRKLCILHRDPGRCYQKIPAFYYNQKKKQCEGFTWESGCGGNSNRFKTIEECRRTCIRK	Kv1.1, 1.2	[57,58]
δDTX	*D. angusticeps*	AAKYCKLPVRYGPCKKKIPSFYYKWKAKQCLPFDYSGCGGNANRFKTIEECRRTCVG	Kv1.1, 1.6	[50,59]
DTX-K	*D. polylepis*	AAKYCKLPLRI GPCKRKIPSFYYKWKAKQCLPFDYSGCGGNANRFKTIEE E CRRTCVG	Kv1.1	[43]
DaE	*D. angusticeps*	LQHRTFCKLPAEPGPCKASIPAFYYNWAAKKCQLFHYGGCKGNANRFSTIEKCRRACVG	Kv1.1	[51]
DV14	*D. viridis*	AAKYCKLP VRYGPCKKKIPSFYYKWKAKQCLPFDYSGCGGNANRFKTIEECRRTCVG	Kv1.1/2	[46]

**Table 2:** The list of protein neurotoxins isolated from snake venom and their Kv channel targets.

Snake Polypeptides	Source	Molecular Mass (kDa)	Potassium Channel Targets	References
**BPTI-Kunitz type**				
DTXs	*Dendroapis angusticeps* *Dendroapis polylepis* *Dendroapis viridis*	7	Kv1.1, Kv1.2, and Kv1.6 antagonists	[43,50,57,58,59]
BF9	*Bungarus fasciatus*	9	Kv1.3 antagonist	[81]
**PLA_2_**				
Crotamine	*Crotalus durissus terrificus*	4.8	Kv1.1, Kv1.2, and Kv1.3 antagonists	[93,106]
β-Bungarotoxin	*Bungarus multicinctus*	22	Inhibition of Kv currents (including Kv1.2 and Kv1.1).	[123,124,135,136,137]
Natratoxin	*Naja atra*	13	A-type Kv channels antagonist	[140]
MiDCA1	*Micrurus dumerilii carinicauda*	15.5	Kv2.1 antagonist	[154,155]
Taipoxin	*Oxyuranus s. scutellatus*	4.6	Inhibition of slowly activating Kv channels	[133,158,159,160]
Notexin	*Oxyuranus s. scutellatus*		Inhibition of slowly activating Kv channels	
Crotoxin	*Crotalus durissus terrificus*	24	Inhibition of slowly activating Kv channels	
**CRISPs**				
BaltCRP	*Bothrops alternatus*	24	Inhibition of Kv1.1, Kv1.3, Kv2.1, and Shaker channels	[165]
Natrin	*Naja atra*	25	Inhibition of the Kv1.3 and BK_Ca_ channels	[163,164]
Stecrisp	*Trimeresurus stejnegeri*	25	Possible Kv channels	[166]
**SVSPs**				
Collinein-1	*Crotalus durissus collilineatus*	29.5	Inhibitor for hEAG1 (Kv10.1) channel (anticancer effect)Mild inhibitor for hERG1 (Kv11.1) channel	[174,175]
Gyroxin_B1.3	*Crotalus durissus terrificus*	28	Inhibitor for hEAG1 (Kv10.1) channel	
BjSP	*Bothrops jararaca*	28	Inhibitor for hEAG1 (Kv10.1) channel	
**Three-finger toxins**				
Cardiotoxin-I	*Naja kaouthia*	6.7	Possible antagonist for K_ATP_ channel involved in stimulating insulin release	[179,182]
Crotalphine	*Crotalus durissus terrificus*	1.5	Possible agonist for K^+^ channels mediating antinociceptive effect	[184,185]

## Data Availability

No new data were created or analyzed in this study. Data sharing is not applicable to this article.
